# Co-Circulation and Genomic Recombination of Coxsackievirus A16 and Enterovirus 71 during a Large Outbreak of Hand, Foot, and Mouth Disease in Central China

**DOI:** 10.1371/journal.pone.0096051

**Published:** 2014-04-28

**Authors:** Weiyong Liu, Shimin Wu, Ying Xiong, Tongya Li, Zhou Wen, Mingzhe Yan, Kai Qin, Yingle Liu, Jianguo Wu

**Affiliations:** 1 State Key Laboratory of Virology and College of Life Sciences, Wuhan University, Wuhan, People's Republic of China; 2 Department of Clinical Laboratory, Wuhan Medical Treatment Center, Wuhan, People's Republic of China; The University of Hong Kong, Hong Kong

## Abstract

A total of 1844 patients with hand, foot, and mouth disease (HFMD), most of them were children of age 1–3-year-old, in Central China were hospitalized from 2011 to 2012. Among them, 422 were infected with coxsackievirus A16 (CVA16), 334 were infected with enterovirus 71 (EV71), 38 were co-infected with EV71 and CVA16, and 35 were infected with other enteroviruses. Molecular epidemiology analysis revealed that EV71 and CVA16 were detected year-round, but EV71 circulated mainly in July and CVA16 circulated predominantly in November, and incidence of HFMD was reduced in January and February and increased in March. Clinical data showed that hyperglycemia and neurologic complications were significantly higher in EV71-infected patients, while upper respiratory tract infection and C-reactive protein were significantly higher in CVA16-associated patients. 124 EV71 and 80 CVA16 strains were isolated, among them 56 and 68 EV71 strains were C4a and C4b, while 25 and 55 CVA16 strains were B1a and B1b, respectively. Similarity plots and bootscan analyses based on entire genomic sequences revealed that the three C4a sub-genotype EV71 strains were recombinant with C4b sub-genotype EV71 in 2B–2C region, and the three CVA16 strains were recombinant with EV71 in 2A–2B region. Thus, CVA16 and EV71 were the major causative agents in a large HFMD outbreak in Central China. HFMD incidence was high for children among household contact and was detected year-round, but outbreak was seasonal dependent. CVA16 B1b and EV71 C4b reemerged and caused a large epidemic in China after a quiet period of many years. Moreover, EV71 and CVA16 were co-circulated during the outbreak, which may have contributed to the genomic recombination between the pathogens. It should gain more attention as there may be an upward trend in co-circulation of the two pathogens globally and the new role recombination plays in the emergence of new enterovirus variants.

## Introduction

Hand, foot, and mouth disease (HFMD) is a common communicable disease that usually affects children, particularly those less than 5 years old [1]. Since the first case was reported in 1957 [2], HFMD has continued to spread globally and is a continuing threat to public health [3]–[10]. In the past few decades, regularly reoccurring outbreaks of HFMD have been relatively centralized in the Asia-Pacific region, including Japan [6], [11], [12], Malaysia [13]–[15], Taiwan [16]–[18], Singapore [19]–[21], Thailand [22], Korea [23], and Hong Kong [24]. Since 2008, epidemic outbreaks of HFMD in China resulted in a total of approximately 3 million cases, including approximately 1500 fatal cases [25]–[33]. Thus, HFMD has already been emerged as an imperative global hazard, not only threatening the health of the children, but also causing tremendous loss and burden to both families and society.

Surveillance study and etiologic analysis demonstrated that HFMD is most commonly caused by human enteroviruses [34]. The Enterovirus genus in the family Picornaviridae consists of 4 species: A, B, C, and D [35], [36]. EV-A species is composed of at least 16 different serotypes, including 11 coxsackievirus serotypes (CVA2, CVA3, CVA4, CVA5, CVA6, CVA7, CVA8, CVA10, CVA12, CVA14, and CVA16) and 5 enteroviruse serotypes (EV71, EV76, EV89, EV90, and EV91) [37]. It has been demonstrated that enterovirus 71 (EV71) and coxsackievirus A16 (CA16) are the principal pathogens of HFMD [38].

EV71 was first described in 1974 during an outbreak with central nervous system complications in California, USA [39], [40] and associated with many outbreaks with wide clinical manifestations [41]–[43]. Since 1997, EV71 outbreaks have gained more attention as there is an upward trend in the prevalence of EV71 globally [16], [19], [44]–[52]. In recent years, many large outbreaks of EV71-associated HFMD with high morbidity and mortality have occurred in the Asia-Pacific region [8], [15], [53]–[57]. In China, large scale EV71 outbreaks associated with acute neurological diseases were occurred in 2008 [25], [58], and since then the outbreak pattern has repeated and exacerbated each year, with increasing morbidity and mortality [27], [59]. It has been confirmed that large HFMD outbreaks with fatal neurological complications that have occurred since 2008 are mainly due to subgenotype C4 of EV71 [26], [60]–[62].

CVA16 was first isolated in South Africa in 1955 [63] and subsequently sequenced in 1994 [64]. CVA16 related to HFMD have been endemic to Southeast Asia and the Pacific region for decades [21], [65]–[69]. The illness caused by CVA16 infection is usually mild [70], but fatal cases associated with CVA16 infection have also been reported [71]–[73].

An important property of enteroviruses is their ability to undergo extensive genetic recombination, which is well known to contribute to genetic variations and evolution of the viruses. Recombination in enteroviruses was first described in 1962 [74], [75], and since then several studies have demonstrated that recombination is a significant and relatively frequent event in circulating enteroviruses and that genetic exchanges could occur both within a given serotype and between different serotypes [76]–[79]. Surveillance data indicated that EV71 and CVA16 can co-circulate during HFMD outbreaks [20], [67], [80], [81]. Such co-circulation may have contributed to the genomic recombination between EV71 and CVA16 [82], [83], which was believed to have led to the emergence of a recombinant EV71 responsible for the large HFMD outbreak in China in 2008 [58]. Complete genome analysis of prototype EV-A indicated that recombination in the nonstructural region has played a role in the evolution of some EV-A prototypes [76]. Phylogenetic analyses of several available sequences of EV71 have shown that recombination occurred between EV71 and coxsackievirus A16 in the nonstructural region [84], [85] and between different subgenotypes of EV71 viruses [86]. Previous studies have been confirmed that the large-scale HFMD outbreaks with fatal neurological complications that have occurred since 2008 are mainly due to subgenotype C4 of EV71, which was identified as a recombination virus with CVA16 in 3D region [58].

In this study, a comprehensive molecular, clinical, epidemiologic, and etiologic survey of an outbreak of HFMD occurred from 2011 to 2012 in Central China was performed. A total of 1844 patients with HFMD from 26 cities or counties in Central China were hospitalized at Wuhan Medical Treatment Center and enrolled in this study. Molecular epidemiology study revealed that among the 1844 cases, 422patients were infected with CVA16, 334 infected with EV71, 38 co-infected with EV71 and CVA16, and 35 infected with other enteroviruses. Among them, 124 EV71 strains, 80 CVA16 strains, and 9 other enterovirus strains were isolated and analyzed. A spatial-temporal surveillance showed that both EV71 and CVA16 were detected year-round, but CVA16 circulated predominantly in November and EV71 circulated mainly in July. Clinical analysis showed that neurologic complications were significantly higher in EV71-infected patients, while the proportion of upper respiratory tract infections (URTIs) and the levels of C-reactive protein (CRP) were significantly higher in CVA16-infected patients. There were no significant differences between EV71-infected group and CVA16-infected group with respect to skin and mouth rashes, bronchitis, leukocytosis, polycythemia, and the concentration of blood leukocytes, erythrocytes, or glucose. Phylogenetic analyses demonstrated that all EV71 isolates belonged to C4a and C4b, and all CVA16 isolates belonged to B1a and B1b. Interestingly, we revealed that recombinant variants of EV71 and CVA16 were identified in 6 isolates, which caused mild and severe HFMD. More importantly, we noticed that EV71 C4b was reemerged and circulated widely during this outbreak after a six-year quiescent period in China.

## Results

### Prevalence of human enteroviruses in a HFMD outbreak in Central China

HFMD, caused by a group of enteroviruses, is a common and rash-associated illness in children under the age of 5, but can lead to mortality in large-scale outbreaks [1]. Since first being described in California [40], there have been many reports on large HFMD outbreaks globally [3], [11], [13], [20], [41], [47], [48], [52], [55], [81]. HFMD has recently been emerged as an imperative hazard, not only threatening the health of the children, but also causing tremendous loss and burden to both families and society in China [30], [32], [67]. Thus, it is necessary to perform a comprehensive survey of HFMD.

During April 2011 to March 2012, a total of 1844 HFMD patients from 26 cities and counties in Central China were diagnosed as HFMD and hospitalized at Wuhan Medical Treatment Center. Throat swabs from each of the patients were collected and subjected to RNA extraction and RT-PCR detection for causative agents using three sets of primers (Table 1), which specifically amplify a universal sequence (EVU) commonly presented in all human enteroviruses, and the specific sequences of EV71 and CVA16, respectively, as described previously [28]. Results showed that among the 1844 HFMD patients, 422 (22.8%) were infected with CVA16, 334 (18.1%) were infected with EV71, 38 (2.1%) were co-infected with EV71 and CVA16, and 35 (1.9%) were infected with other enteroviruses. For the 829 enterovirus-positive specimens, viral isolation was performed. Among them, 124 EV71 strains, 80 CVA16 strains, and 9 other enterovirus genotypes were isolated. Thus, our results demonstrated that 45.0% HFMD patients were infected with enteroviruseses and revealed that CVA16 and EV71 were the main causative agents of this HFMD outbreak in Central China.

**Table 1:** Primers used in this study for PCR amplification and sequencing.

Primer*	Nucleotide sequence (5′→3′)	Location†	Amplico size (bp)	annealing temperature (°C)
PE1	TCCGGCCCCTGAATGCGGCTAATCC	455–479	114	50.0
PE2	ACACGGACACCCAAAGTAGTCGGTCC	543–568		
EV71-S	GCAGCCCAAAAGAACTTCAC	2372–2392	227	57.5
EV71-A	ATTTCAGCAGCTTGGAGTGC	2578–2598		
CA16-S	ATTGGTGCTCCCACTACAGC	2335–2354	209	58.0
CA16-A	TCAGTGTTGGCAGCTGTAGG	2524–2543		
EV71F	GTCCTTAATTCGCACAGCACAGCT	2643–2666	508	58.0
EV71R	CGGTCCGCACTGAGAATGTACCCAT	3126–3150		
CA16F	GGGGATCCCATTGCAGATATG	2446–2466	891	57.5
CA16R	CAACGTTGTTATCTTGTCTCT	3316–3336		
EV71-1-F	CAAGCACTTCTGTTTCCCCGG	167–187	853	54.3
EV71-1-R	GATGGTGGAGTTGCCAATAGTT	998–1019		
EV71-2-F	CACTGAGGGTTCCACCATAAACTAC	800–824	866	52.0
EV71-2-R	CCAATGTGATAGTTATAGGGA	1645–1665		
EV71-3-F	TAGTTGTGCCTATTAGCCCACT	1594–1615	995	55.1
EV71-3-R	AGCAGCTTGGAGTGCTGGAACCTT	2565–2588		
EV71-4-F	AGACGGGCACCATCCAGGGAGA	2422–2443	1071	55.7
EV71-4-R	GGGCAATCGTGTCACAACCT	3473–3492		
EV71-5-F	CATCTTGCCACTCACAATGATT	3390–3411	1044	54.2
EV71-5-R	TCGGTGTTTGCTCTTGAACTG	4413–4433		
EV71-6-F	AGAGTCTATGCCCTGGAGAAGA	4374–4395	823	52.7
EV71-6-R	GAATAATCCAGCCTTGATCCCT	5175–5196		
EV71-7-F	AGTGTAGATAGTGAAGAAGT	5142–5161	1133	49.2
EV71-7-R	TGAAGATCAATAGCCTCAAG	6255–6274		
EV71-8-F	CTCTCAAATGAGCATGGAGGAG	6212–6233	985	54.4
EV71-8-R	GAGGCACAAGGACCGCACAT	7177–7196		
CA16-1-F	CAAGCACTTCTGTTTCCCCGG	171–191	1241	54.8
CA16-1-R	TGTAGTGGCGTAGGGAGGAT	1391–1410		
CA16-2-F	GGGACCGGGAACGAGAAT	1369–1386	1147	54.5
CA16-2-R	GCAACGCAGTCAAGGAGC	2498–2515		
CA16-3-F	AACAATCAAGTGAATCGCTCCT	2482–2503	1172	54.5
CA16-3-R	TCCGAGTGACCCACAGCA	3636–3653		
CA16-4-F	GTTGGGAAAGTTTGGACAGC	3333–3352	1177	53.8
CA16-4-R	AATAATGCCCGTAGCAAGTGA	4489–4509		
CA16-5-F	GAGCAAACACCGTATTGAACC	4428–4448	907	53.7
CA16-5-R	GGCACCTTGAAATCCAGCA	5316–5334		
CA16-6-F	TGCCATCAGTGACTTACTTGCT	5127–5148	1296	53.8
CA16-6-R	TCTTTCACATAGGTGGAATAGGG	6400–6422		
CA16-7-F	AGACTTGCCCTATTCCACCTAT	6393–6414	831	53.4
CA16-7-R	TTCCCATTATGCCACGCTA	7205–7223		

*Primers from EV71-F-165 to EV71-R-7195 are used for genome sequencing of enterovirus 71, and primers from CA16-F-165 to CA16-F-7218 are used for genome sequencing of coxsackievirus A16.

† The location is relative to enterovirus 71 BrCr or coxsackievirus A16 G-10.

### Monthly distributions of HFMD cases and incidence of viral infection

Seasonality in the incidence of HFMD has been reported in several studies, including a seasonal peak of HFMD was detected during summer in Japan [12], [43] and most HFMD cases were reported during autumn in Finland [87]. In Asian regions, HFMD epidemics usually were peaked in the late spring and early summer, along with a second small peak in late autumn and early winter [16], [20], [88], [89]. The seasonality of HFMD suggests that meteorological variables such as mean temperature, relative humidity and wind speed might be influential in the spread of the disease [43], [90]–[92].

**Figure 1:**
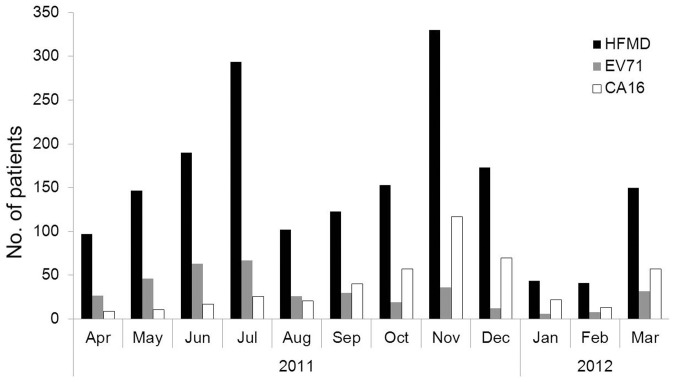
Monthly distributions of patients with HFMD and viral infection in Central China. During April 2011 to March 2012, a total of 1844 HFMD patients from Central China were hospitalized at Wuhan Medical Treatment Center and enrolled in this study. Throat swabs from each of the patients were collected and subjected to RNA extraction and RT-PCR detection using three sets of primers specifically amplify a universal sequence commonly presented in all human enteroviruses, and the specific sequences of EV71, and CVA16, respectively. Among them, 422 were infected with CVA16, 334 were infected with EV71, 38 were co-infected with EV71 and CVA16, and 35 were infected with other enteroviruses. Monthly distribution of the 1844 patients with HFMD, including the 422 patients infected with CVA16, the 334 patients infected with EV71 (EV71), and the 38 patients co-infected with EV71 and CVA16, were described.

In this study, monthly distribution of the total 1844 HFMD cases and the patients infected with EV71 or CVA16 was investigated, respectively. Overall, HFMD cases, EV71-infected patients, and CVA16-infected patients were observed throughout the study period from April 2011 to March 2012 (Figure 1), suggesting that HFMD became endemic year-around in this region. However, two distinct seasonal peaks of HFMD were observed during this period, the first peak occurred from April through August with the highest peak in June and the second peak occurred from September through December with the highest peak in November (Figure 1). More interestingly, distinct monthly distributions between EV71 and CVA16 were also observed in this period. EV71 was the main causative agent accounting for 74.8% (229/306) of EV71- and CVA16-positive cases during the first peak, while CVA16 was the predominant causative agent accounting for 78.7% (284/361) of EV71- and CVA16-positive cases during the second peak (Figure 1). In addition, EV71-associated and CVA16-associated cases were reduced in January and February 2012, only accounting for 6.2% (49/794) of all EV71- and CVA16-positive cases, possibly due to the cold climate during these months. However, HFMD, EV71-positive cases, and CA16-positve cases were increased in March 2012 (Figure 1). These results indicated that the outbreak of EV71- and CVA16-associated HFMD were seasonal dependent. The epidemic season of EV71-associated cases, from May to July, was significantly different from the epidemic season of CA16-associated cases, which ran from October to December (p<0.001).

### Age and gender distribution of HFMD cases and infection of viruses

**Figure 2:**
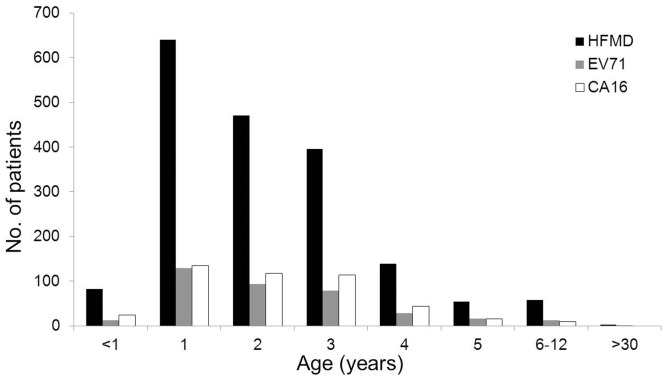
Age distribution of patients with HFMD and viral infection in Central China. During April 2011 to March 2012, a total of 1844 HFMD patients from Central China were hospitalized at Wuhan Medical Treatment Center and enrolled. Among them, 422 were infected with CVA16, 334 were infected with EV71, 38 were co-infected with EV71 and CVA16, and 35 were infected with other enteroviruses. Age distribution of the 1844 patients with HFMD, including the 422 patients infected with CVA16, the 334 patients infected with EV71, and the 38 patients co-infected with EV71 and CVA16, were described.

HFMD usually affects children aged below 10 years, especially those aged below 5 years [11], [22]. In this study, age distribution analyses showed that the most HFMD cases, including EV71-infected and CA16-infected patients, were less than 5 years old (Figure 2). Young children of age 1–3 were the major affected group, with most cases in children of 1–2-year-old. These results suggested that childcare centers were not the major sites for dissemination of this disease. Notably, there was a reduced incidence of HFMD in children less than 1 year-old, presumably due to maternal antibodies and less exposure to other children. For children old than 1-year, the number of EV71-associated and CA16-associated cases decreased visibly as age increased (Figure 2).

Although both children and adults are at risk of infecting enteroviruses, few adult cases of HFMD have been reported. In adults, the transmission of enteroviruses within households is common; however, the manifestation of the viral infection is usually limited to mild illnesses [93]. We identified three adult cases of HFMD and showed that among these incidences, a 33-year-old male who visited the hospital in December 2011 was confirmed to be infected with EV71. These results suggested that the susceptibility of children to EV71- and CA16-associated HFMD is age-dependent and that 1–3-year-old children are the most susceptible.

**Figure 3:**
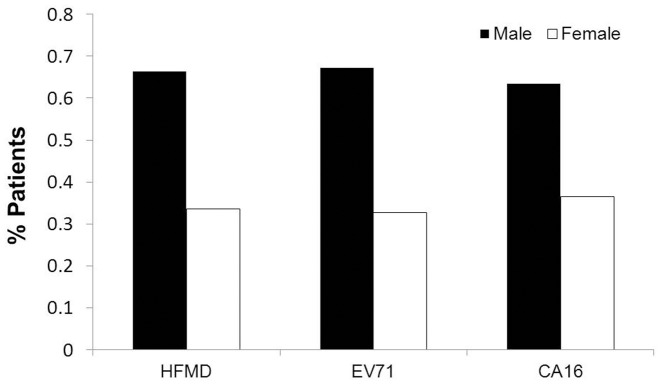
Percentage gender distribution of patients with HFMD in Central China. During April 2011 to March 2012, a total of 1844 HFMD patients from Central China were hospitalized at Wuhan Medical Treatment Center and enrolled. Among them, 422 were infected with CVA16, 334 were infected with EV71, 38 were co-infected with EV71 and CVA16, and 35 were infected with other enteroviruses. Percentage gender distribution of the 1844 patients with HFMD, including the 422 patients infected with CVA16, the 334 patients infected with EV71, and the 38 patients co-infected with EV71 and CVA16, were described.

We also analysis the gender distribution of HFMD cases and infection of viruses. Results showed that among the HFMD cases, 66.3% were male and 33.4% were female (Figure 3). For the EV71-associated cases, the number of male cases was twice (2.0∶1.0) the number of female cases, while for the CVA16 cases, the number of male EV71 cases is almost twice (1.7∶1.0) the number of female cases (Figure 3).

### Clinical features and laboratory data

In most cases, enterovirus infections are generally mild, however, they have also been implicated to cause a variety of diseases, ranging from asymptomatic infection, herpangina, HFMD, to more severe diseases such as menigoencephalitis, myocarditis, and neonatal sepsis, and possibly fatal encephalitis [34], [42], [49].

We carried out a comparison of clinical and laboratory study between EV71-infected patients and CVA16-infected patients in this study. Leukocytosis, polycythemia, and hyperglycemia were defined by a leukocyte count, erythrocyte count, and blood glucose concentration >17.5×10^9^/l, 5.50×10^12^/l, and 8.30 mmol/l on admission, respectively. Our results revealed that body temperature (>38°C), hyperglycemia, vomiting, and central nervous system complications, including limb shaking, lethargy, and convulsion, were significantly more frequent detected in patients with EV71 infection compared to patients with CVA16 infection (Table 2). In contrast, upper respiratory tract infections (URTIs), including pharyngitis and tonsillitis, were significantly more frequent found in CVA16-infected patients compared to EV71-infected patients. The mean serum concentrations of C-reactive protein (CRP) and the proportion of patients with CRP >4.0 mg/l were significantly higher in patients with CVA16 infection than that with EV71 infection (Table 2). We also showed that the percentage of patients with hand rash, mouth rashes, buttock rash, oral rash, were very high (mare than 90%) for both EV71-infected and CVA16-infected patients, but no significant difference between the two groups. In addition, the percentages of patients with bronchitis, leukocytosis, polycythemia, and the concentrations of blood leukocytes, erythrocytes, and glucose were low (less than 10%) for both EV71-infected and CVA16-infected patients, but no significant difference between the two groups (Table 2).

**Table 2:** Comparison of clinical and laboratory data between EV71-infected patients and CVA16-infected patients.

Clinical or laboratory parameter	Enterovirus 71, n = 331	Coxsackievirus A16, n = 410	p value*
Body temperature ≥38°C, no. (%)	243 (73)	212 (52)	<0.001
Hand rash, no. (%)	326 (98)	401 (98)	0.594
Foot rash, no. (%)	324 (98)	398 (97)	0.641
Buttock rash, no. (%)	316 (95)	382 (93)	0.120
Oral rash, no. (%)	298 (90)	379 (92)	0.293
Pharyngitis, no. (%)	54 (16)	127 (31)	<0.001
Tonsillitis, no. (%)	166 (50)	247 (60)	0.007
Bronchitis, no. (%)	26 (8)	35 (9)	0.789
Vomiting, no. (%)	21 (6)	12 (3)	0.031
Limb shaking, no. (%)	14 (4)	6 (1)	0.023
Lethargy, no. (%)	9 (3)	3 (1)	0.041
Convulsion, no. (%)	8 (2)	2 (0.5)	0.049
Leukocyte (10^9^/l), mean (95% CI)	11.32 (10.89–11.75)	11.51 (11.11–11.90)	0.526
Leukocytosis†, no. (%)	31 (9)	35 (9)	0.699
Erythrocyte (10^12^/l), mean (95% CI)	4.65 (4.61–4.69)	4.63 (4.60–4.66)	0.544
Polycythemia‡, no. (%)	10 (3)	5 (1)	0.114
Blood glucose (mmol/l), mean (95% CI)	5.61 (5.32–5.90)	5.37 (5.24–5.50)	0.116
Hyperglycemia§, no. (%)	14 (4)	7 (2)	0.046
CRP¶ (mg/l), mean (95% CI)	1.60 (1.30–1.90)	2.81 (2.31–3.32)	<0.001
CRP>4 mg/l, no. (%)	26 (8)	74 (18)	<0.001

*Contingency data and continuous data were calculated using two-tailed Fisher's exact test and Student's *t*-test, respectively.

† Defined as a leukocyte count >17.5×10^9^/l.

‡ Defined as an erythrocyte count >5.5×10^12^/l.

§ Defined as a blood glucose concentration >8.3 mmol/l.

¶ CRP, C-reactive protein.

### Geographical distribution of HFMD cases in Centre China

Since May 2008, China collected HFMD data in a population-wide manner focused on descriptive epidemiology, seroepidemiology, virology, pathogenesis and treatment aspects, which can be more representative for the actual HFMD epidemic [31], [60], [94]–[96]. A data quality inspection report has revealed that these results are of decent quality, especially in the eastern regions of China [97]. However, there is a need for more frequent statistical analysis of the relationship between geographical location and HFMD incidence in Centre China.

**Figure 4:**
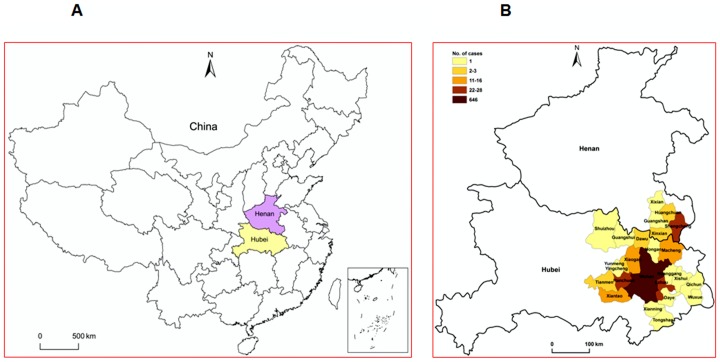
Geographic distribution of HFMD patients infected with CVA16 or EV71 within Henan and Hubei Provinces in Central China. (A) Location of Henan and Hubei Provinces in China. (B) During April 2011 to March 2012, a total of 1844 HFMD patients from Central China were hospitalized at Wuhan Medical Treatment Center and enrolled. Among them, 422 were infected with CVA16, 334 were infected with EV71, 38 were co-infected with EV71 and CVA16, and 35 were infected with other enteroviruses. Geographic distribution of the 422 patients infected with CVA16, the 334 patients infected with EV71, and the 38 patients co-infected with EV71 and CVA16 within Henan and Hubei Provinces in Central China were described. 4 patients are not in the display because they lived far from Wuhan City.

The geographical distribution of HFMD cases due to EV71 or CVA16 infection within Henan and Hubei Provinces of Centre China was investigated in this study. A total of 26 cities or counties in Hubei or Henan Province, Centre China, had confirmed as outbreaks of HFMD due to EV71 or CVA16 infection (Figure 4A). Among the 794 positive cases, Wuhan City had the largest number of EV71-positive and CVA16-positive cases (81.2%, 645/794), followed by Shangcheng County (3.5%, 28/794), Ezhou City (3.3%, 26/794), Hanchuan City (2.8%, 22/794), Xiantao City (2.0%, 16/794), Macheng City (1.9%, 15/794), and Xiaogan City (1.4%, 11/794) (Figure 4B). Our results suggested that the infection rate was highly associated with the population density in urban region.

**Figure 5:**
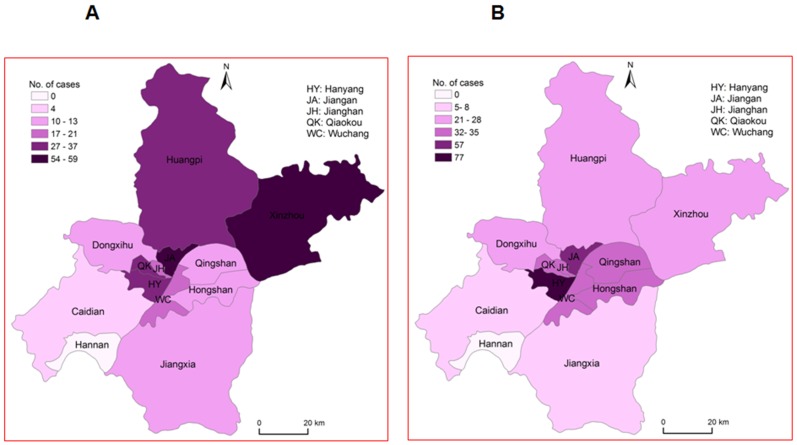
Geographic distribution of HFMD patients infected with CVA16 or EV71 in Wuhan City, China. During April 2011 to March 2012, a total of 1844 HFMD patients from Central China were hospitalized at Wuhan Medical Treatment Center and enrolled. 422 were infected with CVA16, 334 were infected with EV71, 38 were co-infected with EV71 and CVA16, and 35 were infected with other enteroviruses. (A) Geographic distribution of 295 EV71-infected patients in Wuhan City was described. (B) Geographic distribution of 388 CVA16-infected patients in Wuhan City was described.

More detailed analyses of the geospatial distribution within Wuhan City showed that EV71 was the predominant enterovirus in Xinzhou district (59/80, 73.8%) and Jiangxia district (10/15, 66.7%) (Figure 5A), whereas CVA16 was the predominant enterovirus in Qinshan district (32/42, 76.2%), Hongshan district (33/45, 73.3%), Dongxihu district (28/41, 68.3%), Hanyang district (77/114, 67.5%), Wuchang district (35/52, 67.3%), and Caidian district (66.7%, 8/12) (Figure 5B). Other regions, including Jiangan district, Qiaokou district, Jianghan district, and Huangpi district, had high rates of both EV71 and CVA16 infection, but no significant differences between infections of the two viruses (Figure 5A and 5B). It is not clear how geographical location influence HFMD outbreaks, and thus, there is an urgent need to investigate such relationships that could help prediction of future outbreak and evaluation of mitigation strategies.

### Molecular characterization and phylogenetic analyses of EV71 and CVA16 isolates

CVA16 and EV71 are most frequently implicated in HFMD, and other serotypes, including coxsackieviruses A4–10, A24, coxsackieviruses B2–5, and echovirus 18, can also cause HFMD [38]. Based on the capsid protein (VP1) coding region, EV71 is divided into three genotypes: A, B, and C [98] and within the genotypes B and C, there are further subgenotypes, B1–B5 and C1–C5 [26], [99], [100].

To determine the sub-genotypes of the EV71 and CVA16 strains circulating in Centre China during April 2011 to March 2012, we isolated 124 different EV71 strains and 80 different CVA16 strains from the enterovirus-positive specimens enrolled in this study.

It is known EV71 strains were divided into 11 sub-genotypes (A, B1–B5, and C1–C5) based on their VP1 gene sequences. In this study, 37 EV71 strains obtained from GenBank were used as reference genotypes (Table S1 in File S1). It is also known CVA16 strains were divided into two genotypes (A and B) and three sub-genotypes (A, B1, and B2) based on their VP1 gene sequences. In this study, 33 EV71 strains obtained from GenBank were used as reference genotypes (Table S1 in File S1).

**Figure 6:**
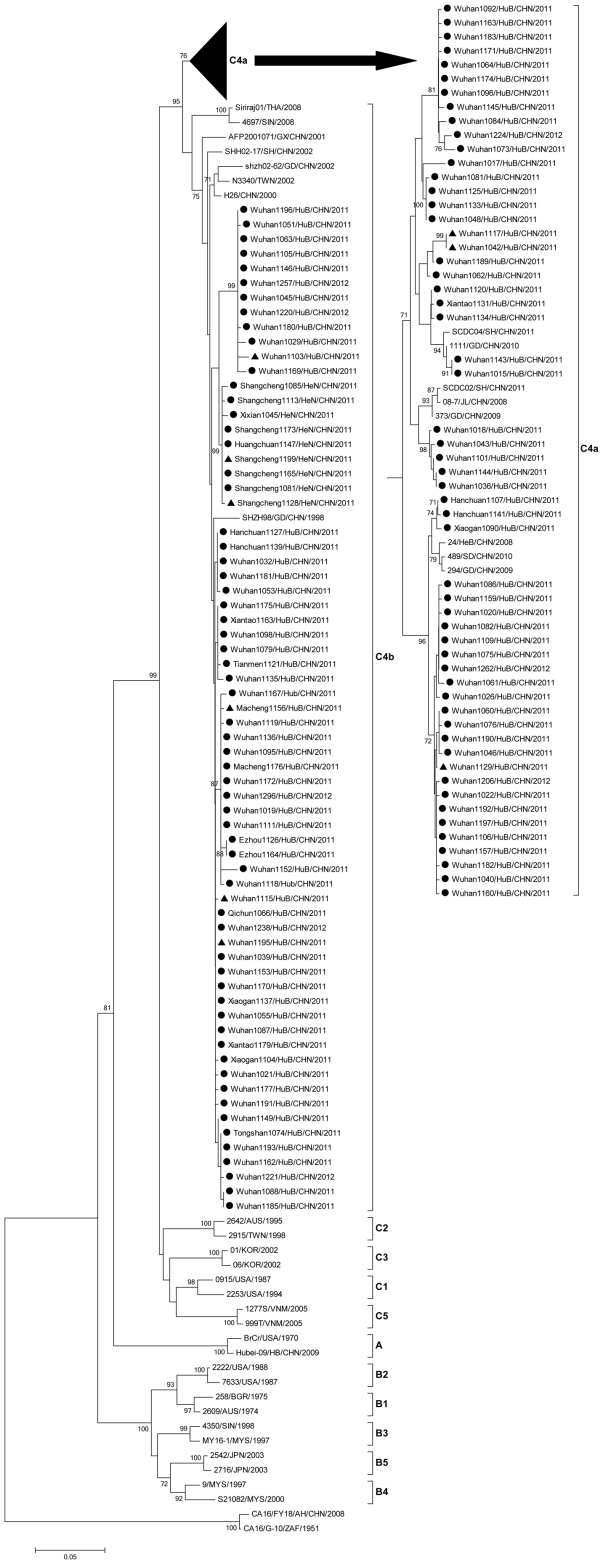
Molecular characterization and phylogenetic analyses of enterovirus 71 (EV71) isolates. Phylogenetic dendrogram constructed by the neighbor-joining method based on 508-bp partial VP1 sequence of the 124 EV71 strains isolated in this study and 38 reference strains. Strains indicated by a filled circle (•) and triangle (▴) are EV71 strains isolated from mild cases and severe cases, respectively, in a HFMD outbreak During April 2011 to March 2012. Bootstrap values with >70 replications are shown at the branch nodes as percentages. The scale bar represents the genetic distance. Strain CVA16 G-10 and CVA16 FY18 strain from China were used as out groups.

The 508-nucleotide of VP1 partial sequence (positions 2643–3150, relative to strain EV71/BrCr) of the 124 EV71 isolates (Table S2 in File S1) were determined and subjected to phylogenetic analyses (Figure 6). Results revealed that the VP1 sequences of all EV71 isolates were clustered with sequences of the EV71 C4 sub-genotype (Figure 6), which has been the dominant circulating EV71 sub-genotype in China since 1998 (It has been reported that subgenotype C4 of EV71 has been the sole viral genetic lineage circulating in mainland China since 1998 [25], [26], [59], [101]. It has been reported that the large HFMD outbreaks with fatal neurological complications that have occurred were mainly due to subgenotype C4a of EV71 [26], [58], [59]. In this study, further phylogenetic analysis showed that 56 EV71 strains and 68 EV71 strains belonged to the C4a and C4b clusters, respectively (Figure 6). EV71 strains isolated from patients in Wuhan, Ezhou, Hanchuan, Xiantao, and Xiaogan belonged to the C4a and C4b clusters, while the EV71 strains isolated from other areas belonged mainly to the C4b cluster (Figure 6). The 56 EV71 C4a strains were isolated from patients in Wuhan, Hanchuan, Xiaotao, and Xiaogan, while 68 EV71 C4b strains were isolated from patients in Wuhan, Shangcheng, Hanchuan, Ezhou, Xiantao, Macheng, Xiaogan, Tianmen, Xixian, Huangchuan, Qichun, and Tongshan (Figure 6). Notably, 9 EV71 C4b strains from Shangcheng, Xixian, and Huangchuan formed a distinct clad (Figure 6), indicating that the EV71 strains circulating in these regions were different from those circulating in Wuhan due to a unique transmission pathway.

**Figure 7:**
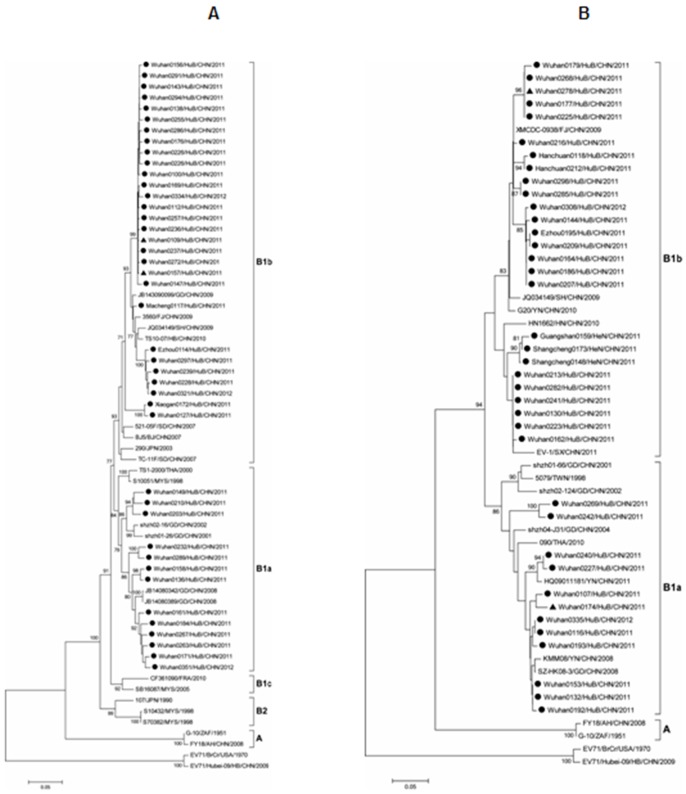
Molecular characterization and phylogenetic analyses of coxsackievirus A16 (CVA16) isolates. (A) Phylogenetic dendrogram constructed by the neighbor-joining method based on 891-bp complete VP1 sequence of the 42 CVA16 strains isolated in this study. (B) Phylogenetic dendrogram constructed by the neighbor-joining method based on 209-bp partial VP3-VP1 sequence of the 38 CVA16 strains. No VP3-VP1 sequences from B1c and B2 reference strains were available from GenBank. Strains indicated by a filled circle (•) and triangle (▴) are CVA16strains isolated from mild cases and severe cases, respectively, during the HFMD outbreak. Bootstrap values with >70 replications are shown at the branch nodes as percentages. The scale bar represents the genetic distance. Strain enterovirus 71 BrCr and another enterovirus 71 Hubei-09 strain from China were used as out groups.

In addition, complete sequences of VP1 gene (891 nucleotides, positions 2446–3336, relative to strain CVA16/G10) from 42 different CVA16 isolates (Figure 7A, Table S3 in File S1) and partial sequences of VP3-VP1 region (209 nucleotides, positions 2335–2543, relative to strain CVA16/G10) of 38 different CVA16 isolates (Figure 7B, Table S4 in File S1) were determined. Phylogenetic analyses showed that all 80 CVA16 strains belonged to the B1 sub-genotype. It is known that B1 sub-genotype of CVA16 can be further classified into three clusters: B1a, B1b, and B1c. We revealed that 25 CVA16 strains belonged to B1a and 55 CVA16strains belonged to B1b. Our results were consistent with previous reports, which showed that almost all CVA16 strains isolated from China from 1999 to 2011 belonged to B1a and B1b clusters [31], [69]. We also showed that the 25 CVA16B1a strains were mainly identified from Wuhan, while the 55 CVA16B1b strains were isolated from Wuhan, Shangcheng, Ezhou, Hanchuan, Xiaogan, Macheng, and Guangshan, suggesting that B1b is more widespread than B1a.

### Genomic recombination between EV71 and CVA16 strains

An important property of enteroviruses is the ability to undergo extensive genetic recombination and viral polymerase-generated mutations, by which the viruses create genetic variation and evolution. Genomic recombination in enteroviruses was originally identified in poliovirus [74], [75]. Since then many studies have revealed that genomic recombination is a significant and relatively frequent event in circulating enteroviruses and that genetic exchanges could occur within a given serotype and between different serotypes [61], [76], [78], [79], [102]–[105].

In this study, phylogenetic analysis revealed that the sequences of three EV71 isolates (two from severe cases and one from mild case) not only shared 77.7%–78.1% identity in nucleotides and 92.0%–92.2% identity in amino acids with EV71 prototype strain BrCr, but also shared 84.6–85.0% homology in nucleotides and 97.4–97.6% homology in amino acids with the CVA16 prototype strain G-10. In addition, phylogenetic analysis also showed that the sequences of three CVA16 isolates (two from severe cases and one from mild case) not only shared 77.7%–78.1% identities in nucleotides and 92.0%–92.2% identities in amino acids with the EV71 prototype strain BrCr, but also shared 84.6–85.0% homology in nucleotides and 97.4–97.6% identity in amino acids with CVA16 prototype strain B1b. To identify the possible genomic recombination events within these strains, the entire genomic sequences of the three EV71 strains (Wuhan1117/HuB/CHN/2011, Wuhan1042/HuB/CHN/2011, Wuhan1143/HuB/CHN/2011) and the three CVA16 strains (Wuhan0109/HuB/CHN/2011, Wuhan0157/HuB/CHN/2011, and Wuhan0127/HuB/CHN/2011) were determined and submitted to GenBank (accession nos. JX986737, JX986738, JX986739, JX986740, JX986741, and JX986742), respectively.

**Figure 8:**
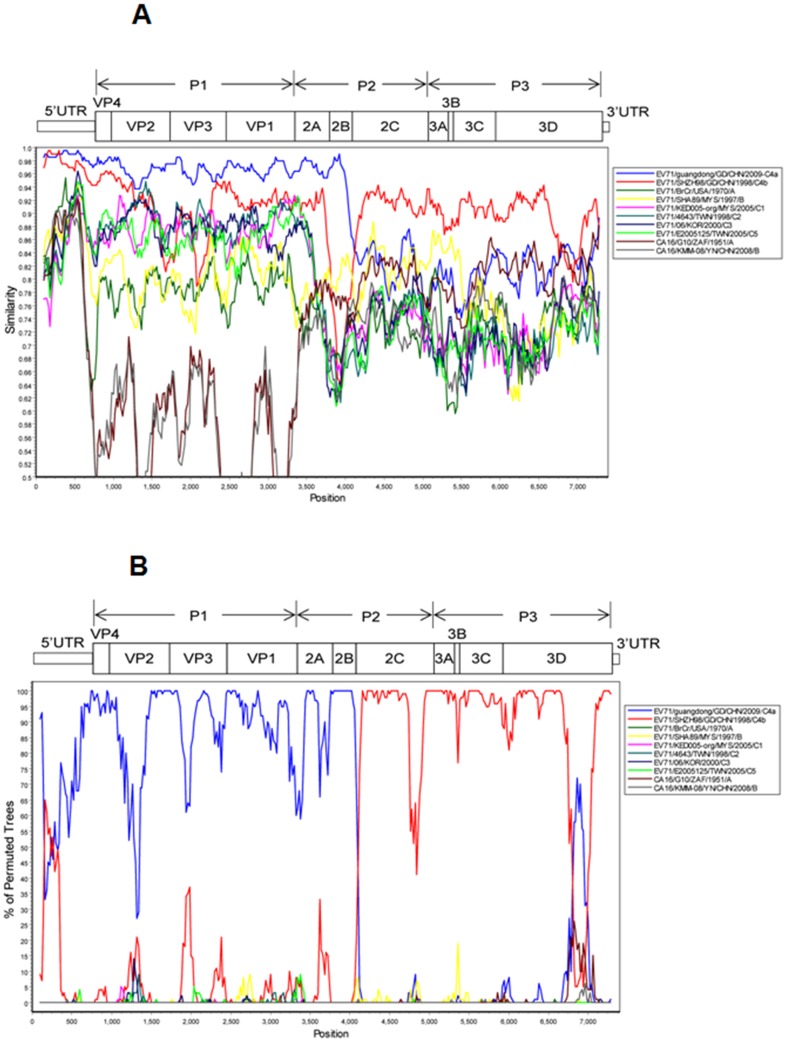
Similarity plot and bootscan analyses of enterovirus 71 (EV71) strain Wuhan1042/HuB/CHN/2011. (A) Similarity plot of EV71 strain Wuhan1042/HuB/CHN/2011 and its potential parent. The y-axis gives the percentage identity in a sliding 200-bp window with 20-bp steps. (B) Bootscan analyses of EV71 strain Wuhan1042/HuB/CHN/2011 and its potential parent. The y-axis gives the percentage of permutated trees in a sliding 200-bp window with 20-bp steps.

Phylogenetic analyses revealed that the three EV71 strains (JX986737, JX986738, and JX986739) belonged to the C4a sub-genotype based on the sequences of VP1. The genome homology between the two EV71 strains (Wuhan1117/HuB/CHN/2011 and Wuhan1143/HuB/CHN/2011) isolated from the two severe cases was 99.7% for nucleotide and 99.6% for amino acid, respectively. The genome homology between the EV71 strain (Wuhan1117/HuB/CHN/2011) isolated from a severe case and the EV71 strain (Wuhan1143/HuB/CHN/2011) isolated from a mild case was 98.1% for nucleotide and 99.3% for amino acid, respectively. Similarity plot graphs (Figure 8A) and bootscan analyses (Figure 8B) based on entire genomic sequences demonstrated that the EV71 strain (Wuhan1042/HuB/CHN/2011, accession no. JX986737) isolated from a severe case had a very high sequence similarity (≥96%) to the EV71 C4a sub-genotype (Guangdong/GD/CHN/2009) in the structural (P1) region and 2A–2B (P2) region, while had a relatively high sequence similarity (≥90%) to the EV71 C4b sub-genotype (SHZH98/GD/CHN/1998) in the 2C (P2) and P3 regions. The most likely breakpoints were located between nucleotides 4034 and 4120, corresponding to the 2B–2C junctions, as determined using the method of maximization of χ^2^ (max χ^2^ = 122.4, p<0.001). Similar results were obtained when EV71 strain (Wuhan1117/HuB/CHN/2011, accession no. JX986738) from a severe case and EV71 strain (Wuhan1143/HuB/CHN/2011, accession no. JX986739) from a mild case were analyzed (data not shown). These results suggested that the three EV71 strains might be recombinant viruses with CVA16 in 2B–2C region.

**Figure 9:**
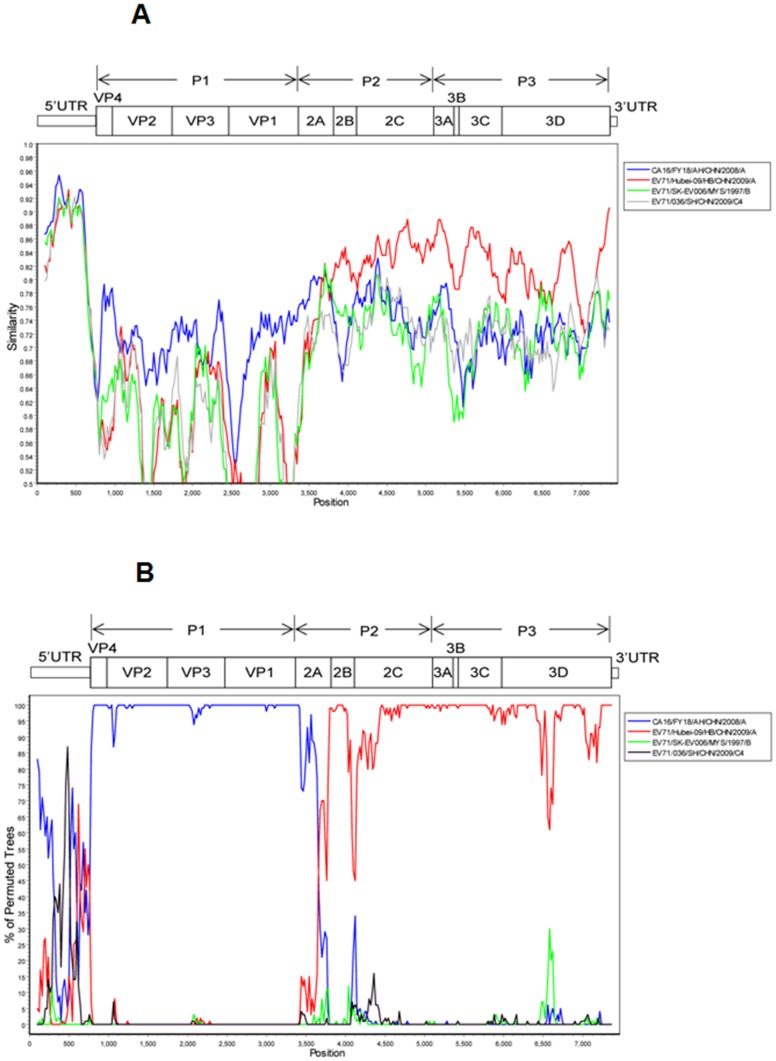
Similarity plots and bootscan analyses of coxsackievirus A16 (CVA16) strain Wuhan0109/HuB/CHN/CHN/2011. (A) Similarity plot of CVA16 strain Wuhan0109/HuB/CHN/2011 and its potential parent. The y-axis gives the percentage identity in a sliding 200-bp window with 20-bp steps. (B) Bootscan analyses of CVA16 strain Wuhan0109/HuB/CHN/2011 and its potential parent. The y-axis gives the percentage of permutated trees in a sliding 200-bp window with 20-bp steps.

Phylogenetic analyses also revealed that the three CVA16 strains belonged to the B1b sub-genotype based on the sequences of VP1. The genome homologies between the two CVA16 strains (Wuhan0109/HuB/CHN/2011 and Wuhan1143/HuB/CHN/2011) from severe cases were 99.7% for nucleotide and 99.5% for amino acid, while the genome homologies between the CVA16 strain (Wuhan1117/HuB/CHN/2011) from a severe case and the CVA16 strain (Wuhan1143/HuB/CHN/2011) from a mild case were (96.2% for nucleotide and 99.0% for amino acid, respectively. Similarity plot graphs (Figure 9A) and bootscan analyses (Figure 9B) based on entire genomic sequences revealed that the CVA16 strain (Wuhan0109/HuB/CHN/2011, accession no. JX986740) from a severe case had a relatively high sequence similarity (≥76%) to the CVA16 A sub-genotype (FY18/AH/CHN/2008) in the structural (P1) and 2A–2B (P2) regions, but had a higher sequence similarity (≥82%) to the EV71 A sub-genotype (Hubei-09/HuB/CHN/2009 2C) in the P2, P3, and 3′UTR regions. The most likely breakpoints were located between nucleotides 3698 and 3734, corresponding to the 2A–2B junctions, as determined using the method of maximization of χ^2^ (max χ^2^ = 69.8, p<0.001). Similar results were obtained when CVA16 strain (Wuhan0157/HuB/CHN/2011, accession no. JX986741) from a severe case and CVA16 strain (Wuhan0127/HuB/CHN/2011, accession no. JX986742) from a mild case were analyzed (data not shown). These results suggested that the three CVA16 strains might be recombinant viruses with EV71 in 2A–2B region.

## Discussion

Since the first case was reported [2], HFMD has continued to spread globally and is a continuing threat to public health [3]–[10]. Regularly reoccurring outbreaks of HFMD have been relatively centralized in the Asia-Pacific region recently [12], [14], [18], [21], [23], [24]. Since 2008, epidemic outbreaks of HFMD in China have resulted in a total of approximately 3 million cases, including approximately 1500 fatal cases [25], [27]–[29], [32], [33].

In this study, we revealed that EV71 and CVA16 were the main causative agents in a large HFMD outbreak during April 2011 to March 2012 in Central China. This result is consistent with previous reports, which demonstrated that HFMD is most commonly caused by human enteroviruses, especially EV71 or CVA16 [34], [38]. More interestingly, we also demonstrated that EV71 and CVA16 were co-circulated in this HFMD outbreak. It has been reported that many large outbreaks of HFMD were only EV71-associated [8], [15], [16], [19], [25], [28], [44], [45], [47], [48], [52]–[54], [57]–[59], [89].

CVA16 related to HFMD have been endemic to Southeast Asia and the Pacific region for decades [21], [66]–[69]. Thus, co-circulation of EV71 and CVA16 during a large outbreak of HFMD in Central China should gain more attention as there may be an upward trend in co-circulation of the two pathogens globally.

We showed that HFMD incidences were observed throughout the study period from April 2011 to March 2012, suggesting that HFMD became endemic year-around in Central China. However, two distinct monthly peaks of HFMD cases were observed, the first one occurred in July and the second one in November. Distinct monthly distributions of EV71-infected and CVA16-infected cases were also observed in the two periods: EV71 was the main causative agent in July, while CVA16 was the predominant causative agent in November. This is the first time that CVA16, rather than EV71, was the main causative agent during an outbreak of HFMD in early winter in Mainland China since 2008. Ongoing surveillance is needed to confirm whether this alternating epidemic pattern of EV71- and CA16-associated HFMD will recur in a regular annual cycle in the future.

In addition, EV71-associated and CVA16-associated HFMD cases were reduced in January and February, possibly due to the cold climate during these months, but increased in March. The epidemic season (from May to July) of EV71-associated HFMD was significantly different from the epidemic season (from October to December) of CVA16-associated HFMD, suggesting that outbreaks of EV71 and CVA16 were seasonal dependent. Seasonality in the incidence of HFMD has been previously reported, including a seasonal peak of HFMD was detected during summer in Japan [12], [43] and a seasonal peak of HFMD was detected during autumn in Finland [87]. In Asian, HFMD epidemics usually peak in the late spring and early summer, along with a second small peak in late autumn and early winter [16], [20], [88], [89]. The seasonality of HFMD suggests that meteorological variables might be influential in the spread of the disease [43], [90]–[92].

HFMD is a common communicable disease that usually affects children aged below 10 years, especially those aged below 5 years [1], [11], [22]. In this study, we showed that the most HFMD patients were less than 5 years old and children of age 1–3-year-old were the major affected group. This group was unlikely in the kindergartens, but most likely stayed at different households, suggesting that kindergarten and childcare centers were not the major sites for dissemination of the disease. It has reported that EV71 transmission rates were high for children among household contacts, including siblings, cousins, parents, grandparents, uncles, and aunts, and EV71-infected adults in family were usually asymptomatic or mild [93]. The one-child policy was strictly enforced in Mainland China, and thus it is possible that adults with asymptomatic infection in the family may be the major infectious source of HFMD in children of 1–3-year-old. Notably, there was a reduced incidence of HFMD in children less than 1 year-old, presumably due to maternal antibodies and less exposure to other children. For children old than 3-year, the number of HFMD cases was decreased as age increased. Gender distribution analysis of HFMD showed that 66.3% were male and 33.6% were female. For the EV71 cases, the ratio of male to female was twice (2.0∶1.0), while for the CVA16 cases, the ratio of male cases to female cases was almost twice (1.7∶1.0), which was similar to previous report [57].

It has been confirmed that large HFMD outbreaks with fatal neurological complications that have occurred since 2008 in China were mainly due to subgenotype C4 of EV71 [26], [60]–[62]. Enterovirus infections are generally mild, however, they have been implicated to cause a variety of diseases, ranging from asymptomatic infection, herpangina, HFMD, to more severe diseases such as menigoencephalitis, myocarditis, and neonatal sepsis, and possibly fatal encephalitis [34], [42], [49]. We showed that fever, hyperglycemia, vomiting, limb shaking, lethargy, and convulsion were significantly more frequent detected in patients infected with EV71 compared to patients infected with CVA16. These results were in agreement with previous studies [21], [23], [106]–[108]. In contrast, the illness caused by CVA16 infection is usually mild [70]. We showed that upper respiratory tract infections were significantly more frequent in CVA16-infected patients compared to EV71-associated patients, but the percentages of patients with bronchitis, leukocytosis, polycythemia, and the concentrations of leukocytes, erythrocytes, and glucose were less than 10% for both EV71 and CVA16 infections. The mean serum concentrations of CRP were significantly higher in patients with CVA16 infection than that with EV71 infection. The higher levels of CRP with lower concentrations of leukocytes in CVA16-infected patients indicated that leukocyte activation, rather than leukocyte redistribution, may occur in patients with CVA16 infection. To some extent, it may explain why most cases of CVA16 infection were mild.

Phylogenetic analyses revealed that all 124 EV71 strains isolated in this study belong to C4 sub-genotype of EV71, which has been the dominant circulating EV71 sub-genotype in China since 1998 [26], [101]. Further phylogenetic analysis showed that 56 EV71 strains and 68 EV71 strains belong to the C4a and C4b clusters, respectively. Previous studies showed that EV71 C4b was the predominant strain from 1998–2004 in Mainland China, and since then EV71 C4a has been the predominant strain [25], [59], [109]. Thus, our results indicated that after a quiet period of approximately six years, EV71 C4b reemerged and caused a large-scale HFMD epidemic in Central China.

All previously characterized CVA16 strains were divided into two genotypes (A and B) and three sub-genotypes (A, B1, and B2) based on their VP1 gene sequences. We showed that all 80 CVA16 strains isolated in this study belonged to the B1 sub-genotype. It has been showed that almost all CVA16 strains isolated from 1999 to 2011 in China belonged to B1a and B1b clusters [31], [69]. Our results revealed that 25 CVA16 strains belonged to B1a and 55 CVA16strains belonged to B1b, indicating that CVA16 sub-genotype B1b was more widespread than B1a during this outbreak.

Phylogenetic analyses revealed that the sequences of three EV71 isolates (two from severe cases and one from mild case) not only share high homology with EV71, but also share high identity with CVA16. Similarly, the sequences of three CVA16 isolates (two from severe cases and one from mild case) not only share high identity with CVA16, but also share high homology with EV71. To identify the possible genomic recombination events within these isolates, the entire genomic sequences of the six strains were determined. Similarity plot graphs and bootscan analyses demonstrated that the three EV71 strains had a very high sequence similarity to EV71 C4a sub-genotype in the P1 and P2 regions, while had a relatively high sequence similarity to the EV71 C4b sub-genotype in the P2 and P3 regions. The most likely breakpoints were located between nucleotides 4034 and 4120, corresponding to the 2B–2C junctions, suggesting that the three EV71 strains were recombinant viruses with CVA16 in 2B–2C region. The three CVA16 strains had a relatively high sequence similarity to the CVA16 A sub-genotype in the P1 and P2 regions, but had a higher sequence similarity to the EV71 A sub-genotype in the P2, P3, and 3′UTR regions. The most likely breakpoints were located between nucleotides 3698 and 3734, corresponding to the 2A–2B junctions, suggesting that the three CVA16 strains were recombinant viruses with EV71 in 2A–2B region. These results suggested that the 2B–2C junction and 2A–2B junction in the non-structural region P2 are likely hot spots for enterovirus recombination.

Enteroviruses are able to undergo extensive genetic recombination, by which the viruses create genetic variation and evolution [74], [75]. Genomic recombination is a significant event in circulating enteroviruses and genetic exchanges could occur within a given serotype and between different serotypes [61], [76]–[79], [102]–[105].

In recent years, several types of EV71 vaccines have been developed to prevent HFMD [110]–[115]. The recombination of CVA16 and widespread epidemic caused by this strain indicates that CVA16 should be included along with EV71 to create a bivalent vaccine in order to provide broader, more effective protection against HFMD.

Thus, the comprehensive epidemiology and virology survey of an HFMD outbreak described in this paper would provide valuable information for improved child care and disease prevention. In addition, this founding would provide insight into developing public-health interventions for the control and prevention of HFMD, especially for reducing the risk of HFMD in high-risk individuals by taking precautions against enterovirus infections.

## Materials and Methods

### Ethics statement

The written informed consents from the parents or guardians of children or minors have been obtained. The study was conducted according to the principles of the Declaration of Helsinki and approved by the Institutional Review Board of the College of Life Sciences, Wuhan University, in accordance with its guidelines for the protection of human subjects.

### Clinical information and specimen collection

Collection of clinical information and specimens was curried out as described previously [28]. Mild cases of HFMD were defined as patients with vesicular lesions on their palms, feet, and mouth, with or without fever, whereas severe cases were defined as HFMD accompanied by neurologic or cardiopulmonary complications [16]. All HFMD cases in this study were hospitalized at the Wuhan Medical Treatment Center (also Wuhan Infectious Diseases Hospital). Several cases had visited the smaller hospitals within their district area before being referred to the Wuhan Medical Treatment Center and some of them had already received antiviral therapy at the smaller hospitals. For each patient, a clinical survey was filled out and a thorough medical exam was administered to evaluate other HFMD-associated symptoms. These surveys were obtained from the hospital and used in this epidemiologic study. Medical practitioners collected throat swabs from each case when applicable. These samples were used in the lab for diagnosis and viral isolation.

### Viral RNA extraction and reverse transcription

Viral RNA extraction and reverse transcription-PCR (RT-PCR) were performed as described previously [28]. Briefly, viral RNA was extracted from primary clinical specimens or infected cell culture supernatants using a High Pure Viral RNA Extraction Kit according to the manufacturer's instructions (Roche, Mannheim, Germany). Reverse transcription was performed using random hexamers and M-MLV reverse transcriptase (Promega, Madison, WI, USA).

### Conventional RT-PCR and enterovirus sequence identification

Information about the primer pairs used for the detection, classification, and full-length genome sequencing are shown in Table 1. The primers used to detect EV71 and CVA16 were described previously [28]. First, pan-enterovirus primers (PE1 and PE2) were used to detect human enterovirus by amplifying a fragment within the 5′ untranslated region (5′UTR), which is highly conserved among all human enteroviruses. The primers EV71-S and EV71-A were used for the detection of EV71, while the primers CA16-S and CA16-A were used for the detection of CVA16. All samples were subjected to PCR using each of the three sets of primers. For the typing and genomic sequencing of the isolates, several primer sets (Table 1) were used. The 5′ and 3′ termini of the viral genome were determined using 5′ and 3′ RACE according to the manufacturer's instructions (Life Technologies, Grand Island, NY). The PCR products were purified using a gel extraction kit (Omega Bio-Tek Inc., Norcross, GA), and cloned into pMD-18T (Takara, Dalian, China). Positive clones were subjected to bi-directional DNA sequencing (Life Technologies).

### Enterovirus isolation

To isolate viruses from the clinical specimens, human rhabdomyosarcoma (ATCC CCL-136), human laryngeal epithelial (ATCC CCL-23), and African green monkey kidney (ATCC CCL-81) cells were grown in Dulbecco's modified Eagle's medium (DMED) (Life Technologies) containing 10% fetal bovine serum (FBS) (Life Technologies). The specimens were concentrated at 6000 × *g* for 5 min, and then 50–100 µl of each concentrates was inoculated into a 25-cm^2^ flask containing confluent cell monolayers. After virus absorption for 2 h at 37°C, 4 ml of DMEM supplemented with 2% heat-inactivated FBS was added to each flask, and the flasks were incubated for 5–7 days at 37°C with 5% CO_2_. Characteristic enterovirus cytopathic effects (CPE) were evaluated microscopically each day, the isolates were harvested when 75% of the cultures showed CPE. The presence of enterovirus in the harvests was confirmed by RT-PCR using human enterovirus-specific primers.

### Phylogenetic analyses

Sequences of EV71 or CVA16 were aligned with reference sequences using the Clustal W program implemented in MEGA5.05 [116]. The reference sequences represented all known EV71 and CVA16 sub-genotypes were obtained from the GenBank database (Table S1). Phylogenetic dendrograms were constructed using the neighbor-joining method with MEGA5.05. The stability of the nodes was tested using the bootstrap method with a bootstrap value of 1000 replicates. Bootstrap values below 70% were hidden.

### Similarity plots and bootscan analyses

Similarity plots and bootscan analyses of the complete genome alignments were performed using the SimPlot software package version 3.5.1 [116]–[118]. Similarity was calculated in each 200-nucleotide window using the Kimura two-parameter distance method with a transition-transversion ratio of 2. The window was advanced successively along the genome alignment in 20-nucleotide increments. The breakpoints were identified using the method of maximization of χ^2^ [119], which was calculated with SimPlot software.

### Data analyses

All statistical analyses were performed using Microsoft Excel 2010 (Microsoft, Redmond, WA) or SPSS software version 13.0 (SPSS Institute, Cary, NC). Differences in proportions and means were evaluated using Fisher's exact test and Student's *t*-test, respectively. A p-value <0.05 was considered statistically significant. Geo-statistical maps were used to characterize the spatial distribution with ArcInfo software version 9.3 (ESRI, Redlands, CA).

## Supporting Information

File S1 Supporting tables.
(DOC)
