# Supplementary material for: Co-Circulation and Genomic Recombination of Coxsackievirus A16 and Enterovirus 71 during a Large Outbreak of Hand, Foot, and Mouth Disease in Central China
Source: PLoS One. 2014 Apr 28;9(4):e96051. doi: 10.1371/journal.pone.0096051 (PMC4002479; doi:10.1371/journal.pone.0096051)
Supplement: File S1 — Supporting tables. (DOC) [file pone.0096051.s001.doc]

**Supporting Information - File S1**

**Table S**1. Entervovirus 71 and coxsackievirus A16 reference strains obtained from GenBank

| Genotype | Strain* | GenBank No. | Genotype | Strain* | GenBank No. |
| --- | --- | --- | --- | --- | --- |
| EV71-A | BrCr/USA/1970 | U22521 | CA16-A | FY18/AH/CHN/2008 | [EU812514](http://www.ncbi.nlm.nih.gov/nucleotide/193795851?report=genbank&log$=nucltop&blast_rank=1&RID=7RYAXEP201N) |
| EV71-A | Hubei-09/HB/CHN/2009 | GU434678 | CA16-A | G10/ZAF/1951 | [U05876](http://www.ncbi.nlm.nih.gov/nucleotide/458298?report=genbank&log$=nucltop&blast_rank=1&RID=7RYD5NZU01N) |
| EV71-B1 | 258/BGR/1975 | AB059814. | CA16-B1a | 090/THA/2010 | JF738003 |
| EV71-B1 | 2609/AUS/1974 | AF135886 | CA16-B1a | 5079/TWN/1998 | AF177911 |
| EV71-B2 | 2222/USA/1988 | AF009540 | CA16-B1a | HQ09011181/YN/CHN/2011 | JQ316639 |
| EV71-B2 | 7633/USA/1987 | AF009534 | CA16-B1a | JB14080342/GD/CHN/2008 | HM776264 |
| EV71-B3 | 4350/SIN/1998 | AF376119 | CA16-B1a | JB14080389/GD/CHN/2008 | HM776265 |
| EV71-B3 | MY16-1/MYS/1997 | AF376073 | CA16-B1a | KMM08/YN/CHN/2008 | HQ423141 |
| EV71-B4 | 9/MYS/1997 | AJ586873 | CA16-B1a | S10051/MYS/1998/B1a | AM292454 |
| EV71-B4 | S21082/MYS/2000 | AF376084 | CA16-B1a | shzh01-26/GD/CHN/2001 | AY895103 |
| EV71-B5 | 2542/JPN/2003 | AB177815 | CA16-B1a | shzh01-66/GD/CHN/2001 | [AY895105](http://www.ncbi.nlm.nih.gov/nucleotide/59896195?report=genbank&log$=nucltop&blast_rank=1&RID=7RY7EXJM01N) |
| EV71-B5 | 2716/JPN/2003 | AB177816 | CA16-B1a | shzh02-124/GD/CHN/2002 | AY895094 |
| EV71-C1 | 0915/USA/1987 | AF009549 | CA16-B1a | shzh02-16/GD/CHN/2002 | AY895100 |
| EV71-C1 | 2253/USA/1994 | AF009544. | CA16-B1a | shzh04-J31/GD/CHN/2004 | AY821796 |
| EV71-C2 | 2642/AUS/1995 | AF135948 | CA16-B1a | SZ-HK08-3/GD/CHN/2008 | GQ279368 |
| EV71-C2 | 2915/TWN/1998 | AF286525 | CA16-B1a | TS1-2000/THA/2000 | AM292477 |
| EV71-C3 | 01/KOR/2002 | AY125966 | CA16-B1b | 290/JPN/2003 | AB465400 |
| EV71-C3 | 06/KOR/2002 | AY125970 | CA16-B1b | 3560/FJ/CHN/2009 | HQ269389 |
| EV71-C5 | 1277S/VNM/2005 | EU072168 | CA16-B1b | 521-05F/SD/CHN/2007 | GQ429222 |
| EV71-C5 | 999T/VNM/2005 | EU051147 | CA16-B1b | BJ5/BJ/CHN2007 | JF317960 |
| EV71-C4a | 08-7/JL/CHN/2008 | HM212453 | CA16-B1b | EV-1/SX/CHN/2011 | JN582179 |
| EV71-C4a | 1111/GD/CHN/2010 | JF519718 | CA16-B1b | G20/YN/CHN/2010 | JN590244 |
| EV71-C4a | 24/HeB/CHN/2008 | HM212447 | CA16-B1b | HN1662/HN/CHN/2010 | JF695009 |
| EV71-C4a | 294/GD/CHN/2009 | [JN579806](http://www.ncbi.nlm.nih.gov/nucleotide/371997519?report=genbank&log$=nucltop&blast_rank=1&RID=7S0D97TS016) | CA16-B1b | JB143090099/GD/CHN/2009 | HM776283 |
| EV71-C4a | 373/GD/CHN/2009 | HQ668327 | CA16-B1b | JQ034149/SH/CHN/2009 | JQ034149 |
| EV71-C4a | 489/SD/CHN/2010 | HQ668182 | CA16-B1b | TC-11F/SD/CHN/2007 | GQ429227 |
| EV71-C4a | SCDC02/SH/CHN/2011 | JQ766124 | CA16-B1b | TS10-07/HB/CHN/2010 | JX068827 |
| EV71-C4a | SCDC04/SH/CHN/2011 | JQ766126 | CA16-B1b | XMCDC-0938/FJ/CHN/2009 | JQ320267 |
| EV71-C4a | Guangdong/GD/2009 | JF799986 | CA16-B1c | CF361090/FRA/2010 | HE573007 |
| EV71-C4b | AFP2001071/GX/CHN/2001 | JQ742002 | CA16-B1c | SB16087/MYS/2005 | AM292476 |
| EV71-C4b | H26/CHN/2000 | AB115493 | CA16-B2 | 107/JPN/1990 | AB465369 |
| EV71-C4b | N3340/TWN/2002 | EU131776 | CA16-B2 | S10432/MYS/1998 | AM292455 |
| EV71-C4b | SHH02-17/SH/CHN/2002 | AY547500 | CA16-B2 | S70382/MYS/1998 | [AM292461](http://www.ncbi.nlm.nih.gov/nucleotide/148287217?report=genbank&log$=nucltop&blast_rank=1&RID=7RZSUPSS013) |
| EV71-C4b | shzh02-62/GD/CHN/2002 | AY895136 | EV71-C4b | 4697/SIN/2008 | HQ285098 |
| EV71-C4b | SHZH98/GD/CHN/1998 | AF302996 | EV71-C4b | Siriraj01/THA/2008 | FJ862992 |

**Table S**2. Partial VP1 gene sequences of 124 entervovirus 71 strains

| EV71 strains* | GenBank No. | EV71 strains | GenBank No. | EV71 strains* | GenBank No. |
| --- | --- | --- | --- | --- | --- |
| Wuhan1075/HuB/CHN/2011 | JX972005 | Wuhan1101/HuB/CHN/2011 | JX972047 | Shangcheng1081/HeN/CHN/2011 | JX972088 |
| Wuhan1170/HuB/CHN/2011 | JX972006 | Wuhan1144/HuB/CHN/2011 | JX972048 | Hanchuan1139/HuB/CHN/2011 | JX972089 |
| Ezhou1164/HuB/CHN/2011 | JX972007 | Wuhan1018/HuB/CHN/2011 | JX972049 | Wuhan1133/HuB/CHN/2011 | JX972090 |
| Wuhan1189/HuB/CHN/2011 | JX972008 | Wuhan1029/HuB/CHN/2011 | JX972050 | Wuhan1162/HuB/CHN/2011 | JX972091 |
| Wuhan1061/HuB/CHN/2011 | JX972009 | Wuhan1129/HuB/CHN/2011 | JX972051 | Wuhan1060/HuB/CHN/2011 | JX972092 |
| Ezhou1126/HuB/CHN/2011 | JX972010 | Shangcheng1128/HeN/CHN/2011 | JX972052 | Hanchuan1127/HuB/CHN/2011 | JX972093 |
| Wuhan1019/HuB/CHN/2011 | JX972011 | Wuhan1073/HuB/CHN/2011 | JX972053 | Wuhan1115/HuB/CHN/2011 | JX972094 |
| Wuhan1032/HuB/CHN/2011 | JX972012 | Wuhan1171/HuB/CHN/2011 | JX972054 | Wuhan1175/HuB/CHN/2011 | JX972095 |
| Wuhan1153/HuB/CHN/2011 | JX972013 | Wuhan1084/HuB/CHN/2011 | JX972055 | Wuhan1040/HuB/CHN/2011 | JX972096 |
| Hanchuan1141/HuB/CHN/2011 | JX972014 | Wuhan1017/HuB/CHN/2011 | JX972056 | Wuhan1046/HuB/CHN/2011 | JX972097 |
| Wuhan1118/Hub/CHN/2011 | JX972015 | Wuhan1183/HuB/CHN/2011 | JX972057 | Wuhan1195/HuB/CHN/2011 | JX972098 |
| Wuhan1172/HuB/CHN/2011 | JX972016 | Wuhan1081/HuB/CHN/2011 | JX972058 | Qichun1066/HuB/CHN/2011 | JX972099 |
| Wuhan1119/HuB/CHN/2011 | JX972017 | Wuhan1163/HuB/CHN/2011 | JX972059 | Wuhan1157/HuB/CHN/2011 | JX972100 |
| Wuhan1109/HuB/CHN/2011 | JX972018 | Wuhan1145/HuB/CHN/2011 | JX972060 | Wuhan1191/HuB/CHN/2011 | JX972101 |
| Wuhan1185/HuB/CHN/2011 | JX972019 | Wuhan1092/HuB/CHN/2011 | JX972061 | Wuhan1262/HuB/CHN/2012 | JX972102 |
| Hanchuan1107/HuB/CHN/2011 | JX972020 | Wuhan1174/HuB/CHN/2011 | JX972062 | Wuhan1220/HuB/CHN/2012 | JX972103 |
| Wuhan1103/HuB/CHN/2011 | JX972021 | Wuhan1053/HuB/CHN/2011 | JX972063 | Wuhan1257/HuB/CHN/2012 | JX972104 |
| Macheng1176/HuB/CHN/2011 | JX972022 | Shangcheng1165/HeN/CHN/2011 | JX972064 | Wuhan1224/HuB/CHN/2012 | JX972105 |
| Wuhan1096/HuB/CHN/2011 | JX972023 | Xixian1045/HeN/CHN/2011 | JX972065 | Wuhan1296/HuB/CHN/2012 | JX972106 |
| Wuhan1095/HuB/CHN/2011 | JX972024 | Wuhan1042/HuB/CHN/2011 | JX972066 | Wuhan1238/HuB/CHN/2012 | JX972107 |
| Wuhan1146/HuB/CHN/2011 | JX972025 | Wuhan1196/HuB/CHN/2011 | JX972067 | Wuhan1206/HuB/CHN/2012 | JX972108 |
| Wuhan1105/HuB/CHN/2011 | JX972026 | Wuhan1048/HuB/CHN/2011 | JX972068 | Wuhan1221/HuB/CHN/2012 | JX972109 |
| Xiantao1163/HuB/CHN/2011 | JX972027 |  |  |  |  |

**Table S3. Complete VP1 gene sequences of 42 coxsackievirus A16 strains**

| CVA16 strains* | GenBank No. | CVA16 strains* | GenBank No. |
| --- | --- | --- | --- |
| Wuhan0321/HuB/CHN/2012 | JX975764 | Wuhan0294/HuB/CHN/2011 | JX975785 |
| Wuhan0351/HuB/CHN/2012 | JX975765 | Wuhan0143/HuB/CHN/2011 | JX975786 |
| Wuhan0334/HuB/CHN/2012 | JX975766 | Wuhan0237/HuB/CHN/2011 | JX975787 |
| Ezhou0114/HuB/CHN/2011 | JX975767 | Wuhan0176/HuB/CHN/2011 | JX975788 |
| Wuhan0171/HuB/CHN/2011 | JX975768 | Wuhan0291/HuB/CHN/2011 | JX975789 |
| Wuhan0263/HuB/CHN/2011 | JX975769 | Wuhan0257/HuB/CHN/2011 | JX975790 |
| Wuhan0161/HuB/CHN/2011 | JX975770 | Wuhan0286/HuB/CHN/2011 | JX975791 |
| Wuhan0184/HuB/CHN/2011 | JX975771 | Wuhan0297/HuB/CHN/2011 | JX975792 |
| Wuhan0267/HuB/CHN/2011 | JX975772 | Wuhan0127/HuB/CHN/2011 | JX975793 |
| Wuhan0239/HuB/CHN/2011 | JX975773 | Wuhan0203/HuB/CHN/2011 | JX975794 |
| Wuhan0255/HuB/CHN/2011 | JX975774 | Wuhan0149/HuB/CHN/2011 | JX975795 |
| Wuhan0156/HuB/CHN/2011 | JX975775 | Wuhan0210/HuB/CHN/2011 | JX975796 |
| Wuhan0169/HuB/CHN/2011 | JX975776 | Wuhan0232/HuB/CHN/2011 | JX975797 |
| Wuhan0236/HuB/CHN/2011 | JX975777 | Wuhan0289/HuB/CHN/2011 | JX975798 |
| Wuhan0272/HuB/CHN/2011 | JX975778 | Wuhan0158/HuB/CHN/2011 | JX975799 |
| Wuhan0147/HuB/CHN/2011 | JX975779 | Wuhan0136/HuB/CHN/2011 | JX975800 |
| Wuhan0152/HuB/CHN/2011 | JX975780 | Wuhan0109/HuB/CHN/2011 | JX975801 |
| Wuhan0112/HuB/CHN/2011 | JX975781 | Macheng0117/HuB/CHN/2011 | JX975802 |
| Wuhan0226/HuB/CHN/2011 | JX975782 | Xiaogan0172/HuB/CHN/2011 | JX975803 |
| Wuhan0138/HuB/CHN/2011 | JX975783 | Wuhan0157/HuB/CHN/2011 | JX975804 |
| Wuhan0100/HuB/CHN/2011 | JX975784 | Wuhan0228/HuB/CHN/2011 | KC109192 |

**Table S**4. Partial VP3-VP1 sequences of 38 coxsackievirus A16 strains

| CVA16 strains* | GenBank No. | CVA16 strains* | GenBank No. |
| --- | --- | --- | --- |
| Wuhan0308/HuB/CHN/2012 | JX975726 | Wuhan0213/HuB/CHN/2011 | JX975745 |
| Wuhan0335/HuB/CHN/2012 | JX975727 | Wuhan0282/HuB/CHN/2011 | JX975746 |
| Wuhan0298/HuB/CHN/2011 | JX975728 | Wuhan0164/HuB/CHN/2011 | JX975747 |
| Wuhan0269/HuB/CHN/2011 | JX975729 | Wuhan0174/HuB/CHN/2011 | JX975748 |
| Guangshan0159/HeN/CHN/2011 | JX975730 | Hanchuan0118/HuB/CHN/2011 | JX975749 |
| Wuhan0153/HuB/CHN/2011 | JX975731 | Hanchuan0212/HuB/CHN/2011 | JX975750 |
| Wuhan0285/HuB/CHN/2011 | JX975732 | Wuhan0241/HuB/CHN/2011 | JX975751 |
| Ezhou0195/HuB/CHN/2011 | JX975733 | Wuhan0130/HuB/CHN/2011 | JX975752 |
| Wuhan0240/HuB/CHN/2011 | JX975734 | Wuhan0192/HuB/CHN/2011 | JX975753 |
| Wuhan0216/HuB/CHN/2011 | JX975735 | Wuhan0162/HuB/CHN/2011 | JX975754 |
| Wuhan0107/HuB/CHN/2011 | JX975736 | Wuhan0177/HuB/CHN/2011 | JX975755 |
| Wuhan0209/HuB/CHN/2011 | JX975737 | Wuhan0186/HuB/CHN/2011 | JX975756 |
| Wuhan0242/HuB/CHN/2011 | JX975738 | Wuhan0179/HuB/CHN/2011 | JX975757 |
| Wuhan0225/HuB/CHN/2011 | JX975739 | Wuhan0278/HuB/CHN/2011 | JX975758 |
| Shangcheng0173/HeN/CHN/2011 | JX975740 | Wuhan0116/HuB/CHN/2011 | JX975759 |
| Wuhan0132/HuB/CHN/2011 | JX975741 | Wuhan0207/HuB/CHN/2011 | JX975760 |
| Wuhan0144/HuB/CHN/2011 | JX975742 | Wuhan0223/HuB/CHN/2011 | JX975761 |
| Wuhan0193/HuB/CHN/2011 | JX975743 | Wuhan0227/HuB/CHN/2011 | JX975762 |
| Shangcheng0148/HeN/CHN/2011 | JX975744 | Wuhan0268/HuB/CHN/2011 | JX975763 |

**Supporting Information File S1 Legends**

**Table S1. Entervovirus 71 and coxsackievirus A16 reference strains obtained from GenBank.** EV71 strains are divided into 11 sub-genotypes (A, B1-B5, and C1-C5) based on their VP1 gene sequences, while CVA16 strains were divided into two genotypes (A and B) and three sub-genotypes (A, B1, and B2) based on their VP1 gene sequences. Here, 37 EV71 strains from obtained from GenBank were used as reference genotypes, and 33 EV71 strains obtained from GenBank were used as reference genotypes. *The following conventions were used to name the strains: strain number/province/CHN/year of collection for Chinese strains, and strain number/three-letter country code/year of collection for international strains.

**Table S2. Partial VP1 gene sequences of 124 entervovirus 71 strains.** The 508-nucleotide of VP1 partial sequence (positions 2643-3150, relative to strain EV71/BrCr) of the 124 EV71 isolates were determined in this study and subjected to phylogenetic analyses. *The following convention was used to name the strains: strain number/province/CHN/year of collection.

**Table S3. Complete VP1 gene sequences of 42 coxsackievirus A16 strains.** The complete sequences of VP1 gene (891 nucleotides, positions 2446-3336, relative to strain CVA16/G10) from 42 different CVA16 isolates were determined in this study and subjected to phylogenetic analyses. *The following convention was used to name the strains: strain number/province/CHN/year of collection.

**Table S4. Partial VP3-VP1 sequences of 38 coxsackievirus A16 strains.** The partial sequences of VP3-VP1 region (209 nucleotides, positions 2335-2543, relative to strain CVA16/G10) of 38 different CVA16 isolates were determined in this study and subjected to phylogenetic analyses. *The following convention was used to name the strains: strain number/province/CHN/year of collection.
